# Predictors of Hepatic Fibrosis in Type 2 Diabetes Patients with Metabolic-Dysfunction-Associated Steatotic Liver Disease

**DOI:** 10.3390/biomedicines12112542

**Published:** 2024-11-07

**Authors:** Joana D’Arc Matos França de Abreu, Rossana Sousa Azulay, Vandilson Rodrigues, Sterffeson Lamare Lucena de Abreu, Maria da Glória Tavares, Flávia Coelho Mohana Pinheiro, Clariano Pires de Oliveira Neto, Caio Andrade, Alexandre Facundo, Adriana Guimarães Sá, Patrícia Ribeiro Azevedo, Ana Gregória Pereira de Almeida, Debora Camelo de Abreu Costa, Rogério Soares Castro, Marcelo Magalhães, Gilvan Cortês Nascimento, Manuel dos Santos Faria, Adalgisa de Souza Paiva Ferreira

**Affiliations:** 1 Service of Endocrinology, University Hospital of the Federal University of Maranhão (HUUFMA/EBSERH), São Luis 65020-070, Brazil; madagloria@yahoo.com.br (M.d.G.T.); clarianoneto@gmail.com (C.P.d.O.N.); alexandrenfacundo@hotmail.com (A.F.); gilvancortes@uol.com.br (G.C.N.); mfaria1949@gmail.com (M.d.S.F.); 2 Research Group in Endocrinology and Clinical and Molecular Metabolism (ENDOCLIM), Sao Luis 65020-070, Brazil; vandilson.rodrigues@ufma.br (V.R.); sterffeson@hotmail.com (S.L.L.d.A.); flaviamohana@gmail.com (F.C.M.P.); dr.caioanascimento@hotmail.com (C.A.); adrianabeckman2024@gmail.com (A.G.S.); patricia.azevedo@ufma.br (P.R.A.); agfpalmeida@gmail.com (A.G.P.d.A.); magalhaes_ms@yahoo.com.br (M.M.); 3 Post-Graduate Program in Adult Health (PPGSAD), Federal University of Maranhão (UFMA), São Luis 65020-070, Brazil; 4 Service of Hepatology, University Hospital of the Federal University of Maranhão (HUUFMA/EBSERH), São Luis 65020-070, Brazil; dcabreucosta@gmail.com (D.C.d.A.C.); rogeriocastro79@hotmail.com (R.S.C.); adalgisa.ferreira@ufma.br (A.d.S.P.F.); 5 Graduate Program in Health Sciences, Center for Biological and Health Sciences, Federal University of Maranhão, São Luís 65080-805, Brazil

**Keywords:** metabolic-dysfunction-associated steatotic liver disease, type 2 diabetes mellitus, epidemiology, fibrosis

## Abstract

Background/Objectives: Approximately 25% of the world’s population and more than 60% of patients with type 2 diabetes (T2D) have metabolic-dysfunction-associated steatotic liver disease (MASLD). The association between these pathologies is an important cause of morbidity and mortality in Brazil and worldwide due to the high frequency of advanced fibrosis and cirrhosis. The objective of this study was to determine the epidemiologic and clinical-laboratory profile of patients with T2D and MASLD treated at an endocrinology reference service in a state in northeastern Brazil, and to investigate the association of liver fibrosis with anthropometric and laboratory measurements. Methods: A cross-sectional study was performed in a specialized outpatient clinic with 240 patients evaluated from July 2022 to February 2024, using a questionnaire, physical examination, laboratory tests, and liver elastography with FibroScan^®^. Results: Estimates showed that women (adjusted OR = 2.69, 95% CI = 1.35–5.35, *p* = 0.005), obesity (adjusted OR = 2.23, 95% CI = 1.22–4.07, *p* = 0.009), high GGT (adjusted OR = 3.78, 95% CI = 2.01–7.14, *p* < 0. 001), high AST (adjusted OR = 6.07, 95% CI = 2.27–16.2, *p* < 0.001), and high ALT (adjusted OR = 3.83, 95% CI = 1.80–8.11, *p* < 0.001) were associated with the risk of liver fibrosis even after adjusted analysis. Conclusions: The study findings suggested that female sex and BMI were associated with an increased risk of liver fibrosis, highlighting the importance of comprehensive evaluation of these patients. In addition, FIB-4 and MAF-5 provided a good estimate of liver fibrosis in our population and may serve as a useful tool in a public health setting with limited resources.

## 1. Introduction

Metabolic-dysfunction-associated steatotic liver disease (MASLD) is a condition characterized by the presence of hepatic steatosis in individuals without a history of significant alcohol consumption, associated with at least one of the five cardiometabolic risk factors that correspond to the components of metabolic syndrome, which are hypertriglyceridemia, low-HDL cholesterol, hyperglycemia, arterial hypertension, and increased waist circumference [1,2,3]. This condition represents one of the most prevalent chronic liver diseases worldwide, and its occurrence is often associated with type 2 diabetes mellitus (T2D), which represents a significant clinical challenge due to the increasing prevalence of these pathologies and the substantial risk of serious complications, including cirrhosis and hepatocellular carcinoma [4,5,6]. Certainly, studies have indeed demonstrated an intrinsic relationship between MASLD and T2D, where up to one-third of diabetic patients have hepatic steatosis, and a significant proportion of these patients develop advanced hepatic fibrosis. The progression to advanced hepatic fibrosis in MASLD is considered the most important prognostic factor directly related to the morbidity and mortality of this disease [6].

Late diagnosis of MASLD, often in advanced stages of liver cirrhosis, increases the risk of developing hepatocellular carcinoma [7]. There is growing recognition of the importance of early assessment of MASLD in all patients with T2D, performed by multidisciplinary teams, aiming to reduce hepatic complications [8].

According to international guidelines, liver biopsy is still considered the gold standard for the diagnosis of liver fibrosis in MASLD [9,10,11]. However, liver biopsy as an invasive method is not without complications and is not recommended for disease severity monitoring [11]. Therefore, it is recommended to use non-invasive tests as a viable approach to stage fibrosis, such as the fibrosis-4 index for liver fibrosis (FIB-4) and the non-alcoholic fatty liver disease (NAFLD) fibrosis score, which can identify patients with advanced stage 3 or 4 fibrosis. Recently, the metabolic-dysfunction-associated fibrosis 5 (MAF-5) score has been introduced as a non-invasive option for identifying the risk of liver fibrosis [12]. These tests provide a widely accessible, effective, and inexpensive non-invasive assessment of liver fibrosis, using these methods in combination with transient elastography (FibroScan^®^) when appropriate, which has become essential in the risk stratification and management of these patients [13,14,15]. Liver elastography (FibroScan^®^) diagnosis liver fibrosis by measuring the speed at which ultrasound waves travel through the liver. The stiffer the liver due to the progression of fibrosis, the higher the wave propagation speed [16].

The study aims to evaluate the clinical-epidemiological profile and correlate hepatic fibrosis by elastography with anthropometric and laboratory measurements of patients with MASLD and T2D treated at a public referral service in the northeastern region of Brazil. Understanding the epidemiological and clinical factors underlying MASLD, especially in populations with more unfavorable sociodemographic conditions and significant racial admixture, still requires more comprehensive investigations.

## 2. Materials and Methods

This was a cross-sectional and analytical study carried out at the Endocrinology Outpatient Clinic of the University Hospital of the Federal University of Maranhão/HUUFMA, admitting patients who met the inclusion criteria and who agreed to participate in the research by reading and signing the Free and Informed Consent Form (FICF), from June 2022 to February 2024. The project was submitted to and approved by the Research Ethics Committee (CAAE: 51633521.2.0000.5086).

All 240 participants in this study had a previous diagnosis of T2D and hepatic steatosis (HE). Inclusion criteria were an age of 18 years or older, HE confirmed by imaging tests, and diagnosis of T2D. Patients with chronic liver disease caused by viral hepatitis (positive serology for hepatitis B or C), excessive alcohol intake, and use of hepatotoxic drugs (glucocorticoids and methotrexate, among others) were excluded.

Patients underwent a clinical-demographic survey through a standardized questionnaire, in which data were collected regarding sex, age, nationality, self-reported skin color/race, duration of T2D (years), income (minimum wage based on the value for the year 2022), diet (guided or not by a nutritionist and adherence), level of physical activity (according to the International Physical Activity Questionnaire (IPAQ classification)), use of other medications, associated diseases, family history, smoking, and alcohol consumption.

The following clinical variables were assessed: weight (kg), height (m), body mass index (BMI; kg/m^2^), abdominal circumference (cm), hip circumference, and waist-to-hip ratio. The WHO classification for BMI was adopted. The abdominal circumference measurement was considered normal if it was ≤90 cm in men and ≤80 cm in women. The waist-to-hip ratio was considered normal if it was ≤0.9 [17].

Laboratory test values were determined according to the following methodologies and reference values: glycated hemoglobin (high-performance liquid chromatography (HPLC): 4.8 to 5.9%), AST (optimized IFCC: men up to 40 U/L and women up to 32 U/L), ALT (modified UV: men up to 41 U/L and women up to 33 U/L), complete blood count (platelet count: 140,000–4,500,000 *p*/mm^3^), albumin (bromocresol green: 3.5 to 5.2 g/dL), total cholesterol (enzymatic/trinder: <200 mg/dL), HDL (dextran/magnesium sulfate/sulfate: low < 40 mg/dL), LDL (calculated by the Friedewald formula: <150 mg/dL), and VLDL (calculated by the Friedewald formula: <150 ng/mL) [18].

Non-invasive tests for detecting advanced fibrosis, such as FIB-4 and NAFLD scores, were performed using the electronic MDCalc medical calculators, equations, scores, and guidelines (https://www.mdcalc.com). Participants were defined as having a low risk of advanced fibrosis if they had FIB-4 < 1.3 (those aged < 65 years) or FIB-4 < 2.0 (those aged ≥ 65 years) [19]. The NAFLD fibrosis score was divided into 3 categories: less than −1.455 (F0–F2), between −1.455 and 0.675 (undefined score), and greater than 0.675 (F3–F4) [20]. In addition, the MAF-5 score was estimated based on an equation composed of waist circumference, body mass index, diabetes, aspartate aminotransferase, and platelet measures [12].

Patients were also evaluated using transient elastography (FibroScan^®^). In this test, liver stiffness is determined by measuring (LSM) < 8 kPa to indicate the absence of significant fibrosis [16]. Liver stiffness measurements determined through liver elastography were used to divide the study population into two groups, classified as follows: group I with EHM and absent significant fibrosis (LSM < 8 kPa) and group II with EHM and the presence of significant fibrosis (LSM greater than or equal to 8 kPa) [3,16].

Data were analyzed using R software version 4.3.3 (R Core Team, 2024) and GraphPad Prism version 10 (GraphPad Software, San Diego, CA, USA). Descriptive statistics were calculated using the absolute frequency, percentage, mean, and standard deviation (±SD). Chi-squared or Fisher’s exact tests were used to compare frequencies between groups based on the detection of liver fibrosis by transient elastography (LSM cut-off of 8 kPa). The unpaired *t*-test and Mann–Whitney test were selected to compare continuous variables between groups based on normality assessment of the sample distribution (Shapiro–Wilk test).

Receiver operating characteristic (ROC) curves were plotted to determine the diagnostic value of FIB-4 and NAFLD for detecting liver fibrosis at the 8 kPa LSM cut-off point in FibroScan. The area under the ROC curve (AUC) was estimated for each of the FIB-4, NAFLD, and MAF-5 scores. The cut-offs of 1.3, 1.45, 2.0, 2.67, and 3.25 for the FIB-4 score [19], along with −1.455 and 0.675 for the NAFLD score [20], were selected for evaluating sensitivity and specificity values. Additionally, MAF-5 cut-offs of 0 and 1 were also chosen for this evaluation [12].

In addition, multivariate logistic regression analysis was used to estimate the adjusted odds ratio (OR) and 95% confidence interval to identify risk factors for the detection of liver fibrosis by FibroScan. Age and variables found to be significant in the univariate analysis were entered as independent variables in the multivariate analysis. The variance inflation factor (VIF) was used to test for collinearity between the independent variables included in the multivariate logistic regression model. A significance level of 5% (*p* < 0.05) was used for all analyses.

## 3. Results

A total of 240 adults with TD2 (86 men and 154 women) were included in the study. Liver fibrosis was detected by transient elastography (LSM cut-off of 8 kPa and fibrosis scores ≥ F2) in 26.7% of participants. Comparative analysis showed a statistically higher frequency of women in the group with liver fibrosis (79.7% versus 58.5%, *p* = 0.002). The prevalence of obesity was also statistically higher in the liver fibrosis group (53.1% versus 34.1%, *p* = 0.008). The other demographic, lifestyle, and clinical data showed no significant differences between the groups. In both groups, the most frequent variables were age over 60 years, brown skin color, and those classified as having a moderately inactive physical activity pattern, and the most frequent comorbidities were dyslipidemia and hypertension (Table 1).

The comparative analysis of body measurements and serum data is shown in Table 2. In men, BMI was statistically higher in the liver fibrosis group (33.9 ± 6.6 versus 29.7 ± 2.7, *p* = 0.027). Hip circumference was also higher in men with liver fibrosis (106.4 ± 8.7 versus 101.2 ± 8.1, *p* = 0.049). In addition, analysis of serum markers showed that the group with liver fibrosis had statistically higher levels of α-fetoprotein (*p* = 0.027), GGT (*p* < 0.001), HDL cholesterol (*p* = 0.039), AST (*p* < 0.001), and ALT (*p* = 0.014).

Table 3 shows the comparative analysis of CAP, FIB-4, NAFLD, and MAF-5 findings between the groups with and without liver fibrosis. More severe steatosis categories were more frequent in the group with LSM higher than 8 kPa (*p* = 0.007). Higher FIB-4 cut-off values were more common in the group with liver fibrosis (51.5% vs. 27.8%, *p* < 0.001). No significant differences were found between the distribution of NAFLD categories and liver fibrosis (*p* = 0.600). In addition, the group diagnosed with liver fibrosis by transient elastography had a higher frequency of MAF-5 score > 1 (78.1% vs. 60.9%, *p* = 0.037).

**Figure 1:**
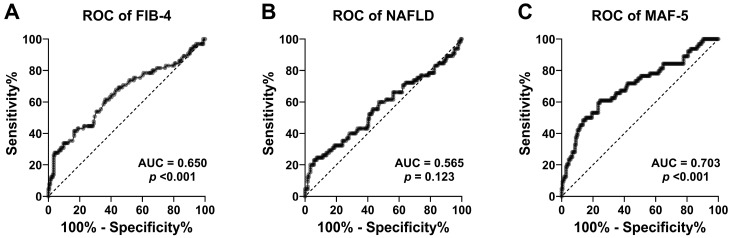
ROC curves of the FIB-4 score (**A**), NAFLD score (**B**), and MAF-5 score (**C**) for the detection of liver fibrosis at the 8 kPa LSM cut-off point in adults with T2D.

Figure 1 and Table 4 show the accuracy assessment of the FIB-4, NAFLD, and MAF-5 scores for the detection of liver fibrosis. The ROC curves showed that the accuracy in predicting liver fibrosis was statistically significant for FIB-4 (AUC = 0.650, 95% CI = 0.566–0.734, *p* < 0.001) and MAF-5 (AUC = 0.703, 95% CI = 0.624–0.782, *p* < 0.001), but not significant for NAFLP (AUC = 0.565, 95% CI = 0.477–0.653, *p* = 0.123). The FIB-4 cut-off of 1.3 exhibited moderate sensitivity (53.8%) and specificity (68.8%). Increasing the FIB-4 cut-off improved specificity but decreased sensitivity. The NAFLD cut-off of −1.455 had high sensitivity (84.6%) but low specificity (13.6%), while the NAFLD cut-off of 0.675 had lower sensitivity (46.2%) but moderate specificity (59.7%). Both MAF-5 cut-offs (0 and 1) had high sensitivity but low specificity.

Multivariate analysis and adjusted ORs are shown in Table 5. The estimates showed that women (adjusted OR = 2.69, 95% CI = 1.35–5.35, *p* = 0.005), obesity (adjusted OR = 2.23, 95% CI = 1.22–4.07, *p* = 0.009), high GGT (adjusted OR = 3.78, 95% CI = 2.01–7.14, *p* < 0.001), high AST (adjusted OR = 6.07, 95% CI = 2.27–16.2, *p* < 0.001), and high ALT (adjusted OR = 3.83, 95% CI = 1.80–8.11, *p* < 0.001) were associated with the risk of liver fibrosis even after adjusted analysis.

Table 6 shows the VIF for the independent variables used in the logistic regression model. The highest VIF values were for high AST and high ALT, but all VIF values were less than 2, indicating that there was no multicollinearity.

## 4. Discussion

The present study assessed liver fibrosis in patients with T2D and MHE, demonstrating that 26.6% of patients assessed by FibroScan^®^ transient elastography (TE) had liver fibrosis (LSM ≥ 8 kPa or ≥F2). Among the patients who developed hepatic fibrosis, 79.7% were female and 53.1% were obese, with significant associations between female sex and BMI in increasing the risk of hepatic fibrosis, highlighting the importance of comprehensive assessment in this patient profile. Most patients in our sample were female, elderly, and of mixed race, reflecting the predominant demographic characteristics of the study population. Furthermore, we emphasized that using the FIB-4 score was a useful tool in assessing the risk of liver fibrosis in a population with low HDI and few public health system resources.

In 2023, several multidisciplinary medical societies developed the new nomenclature, MASLD (metabolic-associated steatotic liver disease), with the aim of appropriately attributing a metabolic basis to this liver disease, which has long been recognized as the “hepatic manifestation of the metabolic syndrome” [21,22]. In this study, the most frequent comorbidities observed were dyslipidemia and arterial hypertension, important components of metabolic syndrome, being compatible with the interrelated pathophysiology between the components of metabolic syndrome and MASLD [23,24].

We observed that all patients were overweight or obese when assessed by BMI. Obesity is considered a major public health threat by the U.S. Department of Health and the World Health Organization (WHO) and is associated with an increased prevalence of multiple chronic conditions, especially metabolic syndrome, compared to individuals without obesity [25,26]. A meta-analysis documented a prevalence of MASLD of 75% in the obese population [27]. Higher BMI was consistently associated with an increased risk of liver fibrosis in our study, and in the literature, it is well established that the risk of developing MASLD is approximately two-fold higher in the setting of obesity compared to healthy, non-obese patients [27,28].

The distribution of android body fat, characterized by increased truncal subcutaneous fat and visceral fat, confers a greater risk of insulin resistance, CVD, and hepatic fibrosis, regardless of BMI [29]. We observed that the waist/height index in our patients was altered (greater than 0.5) [30], both in the group that developed hepatic fibrosis and in those with MASLD without fibrosis, showing that in both groups evaluated with T2D and MASLD, there was a greater disposition of android fat. Patients underwent ET, allowing a non-invasive and accurate assessment of liver fibrosis.

Our results demonstrated a significant association between female sex and higher liver fibrosis (≥F2), suggesting a possible female predisposition to progression to liver fibrosis in MASLD. An epidemiological study in Japan found that the prevalence in women increased with age, especially in postmenopausal women [31], suggesting that hypoestrogenism may play a role in the pathogenesis of MASLD, possibly due to changes in body composition [32]. A cross-sectional cohort study investigating the prevalence of MASLD in 197 pre- and post-menopausal Mexican women demonstrated the disease in 47.2% of women, 32.2% of whom were pre-menopausal and 57.9% of whom were post-menopausal. The authors suggested that estrogen has a protective role in MASLD in women [33]. The mean age of the women in our study was 60 years, which corresponds to the post-menopausal period, corroborating the findings in the literature [32,33]. The comparative analysis showed a statistically higher frequency of women in the group with liver fibrosis, highlighting the importance of differential gender assessment in risk stratification and management of MASLD.

It is known that physical exercise alone, regardless of weight loss, can reduce hepatic steatosis, but histological improvement has not yet been proven [34]. Le et al. [35] gathered data from 5716 individuals and observed that South America had the highest estimated prevalence of MASLD among the continents, with 35.7%. Latin America was ranked as the top region for physical inactivity, with one-third of the population experiencing a lack of physical activity, which we also observed in our patients, where the majority had a pattern of physical inactivity [36].

Regarding clinical scores, higher FIB-4 results were more common in the group with liver fibrosis (51.5% vs. 27.8%, *p* < 0.001), which is consistent with the literature [14,15,21]. Liver biopsy for the diagnosis of fibrosis, despite being considered the gold standard, is a procedure with risks and has important limitations, such as the cost of the procedure, the possibility of sampling errors, morbidity, and non-acceptance by patients due to its invasive nature [10]. Therefore, the use of non-invasive tests to assess the presence of fibrosis is increasingly acceptable in clinical practice [37,38,39]. The fibrosis risk scores, FIB-4 and NAFLD, have proven to be effective tools in identifying patients with MHE, especially FIB-4, which in our study, and most of the literature, showed a correlation with marked hepatic fibrosis [37,38]. This score, together with ET by FibroScan^®^, offers a non-invasive and accessible approach to stage hepatic fibrosis and guide the best clinical management of these patients [40,41,42]. Altered FIB-4 fibrosis risk scores showed a statistically significant association with the risk of hepatic fibrosis in our patients, highlighting the importance of these scores as tools for assessing the risk of fibrosis in our population with liver disease.

In the present study, no significant differences were found between the altered NAFLD score category and liver fibrosis. ROC curves showed that the accuracy in predicting liver fibrosis was statistically significant for FIB-4 but not significant for NAFLD. In our population, FIB-4 performed better than the NAFLD score. The FIB-4 test has been widely used to predict liver fibrosis in patients with chronic liver disease of different etiologies, while the NAFLD score was originally developed to predict liver fibrosis in a biopsy-confirmed cohort [43]. The NAFLD score uses the serum albumin level, which is associated with liver functional reserve, and BMI and plasma glucose as additional metabolic factors. Thus, the FIB-4 test is simpler, with frequently requested variables during screening tests, and has a high negative predictive value [39,40,42], and it was useful for screening liver fibrosis in our population.

The MAF-5 score is a promising and cost-effective approach for assessing liver fibrosis, contributing significantly to the optimization of referrals and resource allocation in healthcare systems [12,43]. By combining anthropometric measurements with key clinical variables, such as diabetes and liver enzyme levels, the MAF-5 stands out, especially in populations under 35 or over 65 years of age. In these age groups, traditional indices, such as FIB-4 and the NAFLD score, which rely heavily on age, may under- or over-estimate fibrosis, leading to less accurate assessments. The MAF-5 has been widely used to estimate liver fibrosis in the general population [20]. In this study, we specifically applied the MAF-5 to assess the risk of liver fibrosis in patients with type 2 diabetes and demonstrated a significant correlation between the MAF-5 scores and the results obtained by elastography, highlighting the accuracy of this index in this population.

In the multivariate analysis of this study, the estimates showed that being female, obese, and having elevated transaminases and gamma GT were associated with the risk of hepatic fibrosis, even after adjusted analysis. Plasma aminotransferases are the most common tests for evaluating MASLD, although they are not as sensitive for the diagnosis of this pathology [23,24]. It is important to note that up to 75% of patients with MASLD may have normal transaminases [24,43]. However, the literature shows that in those patients with altered liver enzymes, there is a greater risk of hepatic fibrosis [24,43], which was also demonstrated in our study, where the elevation of liver enzymes and gamma GT levels showed a strong correlation with the development of hepatic fibrosis.

The strengths of this study include a comprehensive evaluation of a sample of patients from northeastern Brazil with type 2 diabetes and MASLD. This provided robust epidemiologic and clinical insights specific to this population. The use of non-invasive diagnostic tools, such as FibroScan^®^, MAF-5 score, and FIB-4 score, enhanced the practicality and applicability of the findings in resource-limited settings, particularly considering the challenges associated with invasive liver biopsies. Additionally, the focus on key demographic factors—especially the increased risk associated with women and obesity—highlighted important clinical implications for targeted interventions. However, some limitations should be noted, as follow: the cross-sectional design limited causal inference, and the higher proportion of female participants may affect the generalizability of the results. Future studies should aim to validate these findings in larger, more diverse populations, incorporate lifestyle assessments, and develop targeted strategies for the prevention and treatment of MASLD.

## 5. Conclusions

This study provided a comprehensive analysis of the clinical and epidemiologic profiles of patients with T2DM and MASLD treated at a university hospital in Maranhão, a low Human Development Index (HDI) state in northeastern Brazil. Our analysis showed significant associations between female sex and BMI in the increased risk of liver fibrosis, highlighting the importance of comprehensive assessment of these patients to promote weight loss, especially in the menopausal female population. The present findings suggested that FIB-4 and MAF-5 provided a good estimate of liver fibrosis in our population and may serve as useful tools in a public health setting with limited resources. Future research is needed to validate our results in larger populations with a low HDI and to explore intervention strategies for the prevention and treatment of MASLD.

## Figures and Tables

**Table 1:** Demographic, lifestyle, and clinical data of adults with T2D by liver fibrosis diagnosis.

Variables	Diagnosis of Liver Fibrosis by Transient Elastography				*p*
	No (<F2) (*n* = 176, 73.3%)		Yes (≥F2) (*n* = 64, 26.7%)		
Sex, *n* (%)					0.002 *
Male	73	(41.5)	13	(20.3)	
Female	103	(58.5)	51	(79.7)	
Age (in years), mean ± SD	60.3	±9.9	61.5	±8.6	0.606
Age group, *n* (%)					0.390
Up to 40 years old	8	(4.5)	0	(0)	
41 to 50 years old	18	(10.2)	7	(10.9)	
51 to 60 years old	55	(31.3)	21	(32.8)	
Over 60 years old	95	(54.0)	36	(56.3)	
Self-reported skin color/race, *n* (%)					0.842
White	25	(14.2)	10	(15.6)	
Black	36	(20.5)	11	(17.2)	
Brown	115	(65.3)	43	(67.2)	
Smoking, *n* (%)					0.899
Never	101	(57.4)	35	(54.7)	
Former	73	(41.5)	28	(43.8)	
Current	2	(1.1)	1	(1.5)	
Physical activity					0.336
Inactive	63	(35.8)	30	(46.9)	
Moderately inactive	81	(46.0)	27	(42.2)	
Moderately active	28	(15.9)	7	(10.9)	
Highly active	4	(2.3)	0	(0)	
Dietary adherence					0.250
No (<80%)	101	(57.4)	42	(65.6)	
Yes (≥80%)	75	(42.6)	22	(34.4)	
Time since type 2 diabetes diagnosis (in years), mean ± SD	12.5	±8.9	11.9	±9.3	0.637
Comorbidities					
Arterial hypertension	125	(71.0)	45	(70.3)	0.915
Dyslipidaemia	163	(92.6)	59	(92.2)	0.912
Obesity ^1^	60	(34.1)	34	(53.1)	0.008 *
Neuropathy	69	(39.2)	23	(35.9)	0.645
Thyroid dysfunction	43	(24.4)	17	(26.6)	0.736
Osteopenia/osteoporosis	32	(18.2)	15	(23.4)	0.364
Retinopathy	31	(17.6)	8	(12.5)	0.342
Chronic kidney disease	23	(13.1)	4	(6.3)	0.170
Coronary artery disease	17	(9.7)	6	(9.4)	0.947

T2D = type 2 diabetes. ±SD = standard deviation. ^1^ Obesity was defined as body mass index (BMI) ≥ 30 kg/m^2^. F2 = liver stiffness measurement (LSM) ≥ 8 kPa. * *p* < 0.05.

**Table 2:** Anthropometric and serum data of adults with T2D by liver fibrosis diagnosis.

Variables	Diagnosis of Liver Fibrosis by Transient Elastography				*p*
	No (<F2)		Yes (≥F2)		
	Mean	±SD	Mean	±SD	
Anthropometric data (male group)					
BMI (in kg/m^2^)	29.7	±4.3	33.9	±6.6	0.027 *
NC (in cm)	40.1	±2.7	41.7	±3.2	0.085
WC (in cm)	104.4	±11.3	112.2	±15.3	0.105
HC (in cm)	101.2	±8.1	106.4	±8.7	0.049 *
Waist-to-hip ratio	1.03	±0.07	1.05	±0.07	0.400
Waist-to-height ratio	0.63	±0.07	0.68	±0.09	0.130
Anthropometric data (female group)					
BMI (in kg/m^2^)	31.1	±4.7	33.3	±6.7	0.096
NC (in cm)	35.3	±2.5	36.0	±2.9	0.299
WC (in cm)	100.6	±9.7	104.2	±12.8	0.152
HC (in cm)	109.7	±10.6	108.9	±13.5	0.231
Waist-to-hip ratio	0.95	±0.05	0.95	±0.05	0.489
Waist-to-height ratio	0.66	±0.06	0.69	±0.08	0.173
Serum data					
Fasting blood glucose (mg/dL)	154	±69	147	±59	0.779
HbA1c (%)	8.2	±1.8	8.1	±1.9	0.734
Insulin (µIU/L)	22.5	±31.4	20.8	±18.4	0.199
HOMA-IR	4.3	±4.2	4.7	±3.2	0.198
α-fetoprotein (ng/dL)	2.0	±1.1	2.3	±1.2	0.027 *
Ferritin (ng/dL)	152	±158	167	±169	0.418
GGT (U/L)	47.3	±55.7	84.9	±150.5	<0.001 *
Albumin (mg/dL)	4.4	±0.3	4.3	±0.2	0.150
Hemoglobin (g/dL)	13.6	±1.5	13.4	±1.2	0.357
Platelet count (×10^9^/L)	262	±85	245	±87	0.117
Total cholesterol (mg/dL)	163	±44	158	±41	0.737
HDL cholesterol (mg/dL)	43.9	±10.6	47.1	±10.6	0.039 *
LDL cholesterol (mg/dL)	85.4	±37.3	82.6	±35.8	0.790
VLDL cholesterol (mg/dL)	33.7	±19.1	29.6	±13.4	0.240
Non-HDL cholesterol (mg/dL)	116	±42	110	±38	0.519
TG (mg/dL)	169	±99	147	±67	0.243
AST (U/L)	21.1	±11.4	28.9	±18.3	<0.001 *
ALT (U/L)	23.4	±12.6	29.5	±18.0	0.014 *

T2D = type 2 diabetes. ±SD = standard deviation. F2 = liver stiffness measurement (LSM) ≥ 8 kPa. BMI = body mass index. NC = neck circumference. WC = waist circumference. Hb1Ac = glycated hemoglobin. HOMA-IR = homeostasis model assessment of insulin resistance. GGT = gamma glutamyl transferase. HDL = high-density lipoprotein. LDL = low-density lipoprotein. VLDL = very-low-density lipoprotein. TG = triglyceride. AST = aspartate transaminase. ALT = alanine transaminase. * *p* < 0.05.

**Table 3:** Association of features from CAP, FIB-4, NAFLD, and MAF-5 scores with liver fibrosis diagnosed by transient elastography.

Variables	Diagnosis of Liver Fibrosis by Transient Elastography				*p*
	No (<F2)		Yes (≥F2)		
	*n*	(%)	*n*	(%)	
CAP score					0.007 *
No steatosis (S0)	16	(9.1)	0	(0)	
Mild steatosis (S1)	37	(21.1)	7	(10.9)	
Moderate steatosis (S2)	57	(32.7)	22	(34.4)	
Severe steatosis (S3)	65	(37.1)	35	(54.7)	
FIB-4 score					<0.001 *
Lower cut-off value	127	(72.2)	31	(48.4)	
Higher cut-off value	49	(27.8)	33	(51.6)	
NAFLD fibrosis score					0.600
Less than −1.455 (F0–F2)	26	(14.8)	9	(14.1)	
Between −1.455 and 0.675 (Undefined)	80	(45.5)	25	(39.1)	
Above 0.675 (F3–F4)	70	(39.7)	30	(46.8)	
MAF-5 score					0.037 *
<0	31	(17.6)	4	(6.3)	
0–1	36	(20.5)	10	(15.6)	
>1	109	(61.9)	50	(78.1)	

F2 = liver stiffness measurement (LSM) ≥ 8 kPa. CAP = controlled attenuation parameter. FIB-4 = fibrosis-4 score. The cut-off value for FIB-4 was 1.3 for individuals under the age of 65 and 2.0 for those aged 65 and above. NAFLD = non-alcoholic fatty liver disease. MAF-5 = metabolic-dysfunction-associated fibrosis. * *p* < 0.05.

**Table 4:** Sensitivity and specificity measures of the fibrosis-4 (FIB-4) score, the non-alcoholic fatty liver disease (NAFLD) score, and the metabolic-dysfunction-associated fibrosis 5 (MAF-5) score for detecting liver fibrosis at the 8 kPa LSM cut-off point in adults with T2D.

Scores	Cut-Off	Sensitivity	(95% CI)	Specificity	(95% CI)
FIB-4	>1.3	53.8%	(41.9–65.4)	68.8%	(61.6–75.1)
	>1.45	44.6%	(33.2–56.7)	76.7%	(69.9–82.3)
	>2.0	27.7%	(18.3–39.6)	96.0%	(92.0–98.1)
	>2.67	12.3%	(6.4–22.5)	98.3%	(95.1–99.5)
	>3.25	7.7%	(3.3–16.8)	98.9%	(96.0–99.8)
NAFLD	>−1.455	84.6%	(73.9–91.4)	13.6%	(9.3–19.5)
	>0.675	46.2%	(34.6–58.1)	59.7%	(52.3–66.7)
MAF-5	>0	92.2%	(83.0–96.6)	17.6%	(12.7–23.9)
	>1	78.1%	(66.7–86.5)	38.1%	(32.2–45.4)

95% CI = 95% confidence interval.

**Table 5:** Multivariate logistic regression analysis of risk factors for the detection of liver fibrosis at the 8 kPa LSM cut-off point (fibrosis score ≥ F2) in adults with T2D.

Factors	Adjusted OR	(95% CI)	*p*
Age	1.02	(0.98–1.05)	0.197
Female	2.69	(1.35–5.35)	0.005 *
Obesity	2.23	(1.22–4.07)	0.009 *
High α-fetoprotein	1.26	(0.58–2.71)	0.549
High GGT	3.78	(2.01–7.14)	<0.001 *
Low HDL	0.74	(0.39–1.41)	0.372
High AST	6.07	(2.27–16.2)	<0.001 *
High ALT	3.83	(1.80–8.11)	<0.001 *

T2D = type 2 diabetes. OR = odds ratio. 95% CI = 95% confidence interval. GGT = gamma glutamyl transferase. HDL = high-density lipoprotein. AST = aspartate transaminase. ALT = alanine transaminase. * *p* < 0.05.

**Table 6:** Collinearity diagnostics of the independent variables included in the multivariate logistic regression analysis.

Independent Variables	Collinearity Statistics	
	VIF	Tolerance
Age	1.06	0.946
Female	1.06	0.947
Obesity	1.11	0.901
High α-fetoprotein	1.03	0.973
High GGT	1.29	0.773
Low HDL	1.05	0.952
High AST	1.45	0.691
High ALT	1.56	0.640

VIF = variance inflation factor.

## Data Availability

The datasets used and analyzed during the current study are available from the corresponding author upon reasonable request.
